# Supplementary material for: Sorting endosomes play key roles in presentation of Mycobacterium tuberculosis-derived ligands to MAIT cells
Source: Sci Rep. 2026 May 29;16:25556. doi: 10.1038/s41598-026-50843-1 (PMC13478399; doi:10.1038/s41598-026-50843-1)
Supplement: Supplementary file 1 [file 41598_2026_50843_MOESM1_ESM.pdf]

Title

Sorting endosomes play key roles in presentation of *Mycobacterium tuberculosis*-derived ligands to MAIT cells

Authors

Allison E. Tammen[1], Jessie C. Peterson[2], Aneta Worley[2], David M. Lewinsohn[2,3], Elham Karamooz[2,3*]

*corresponding author

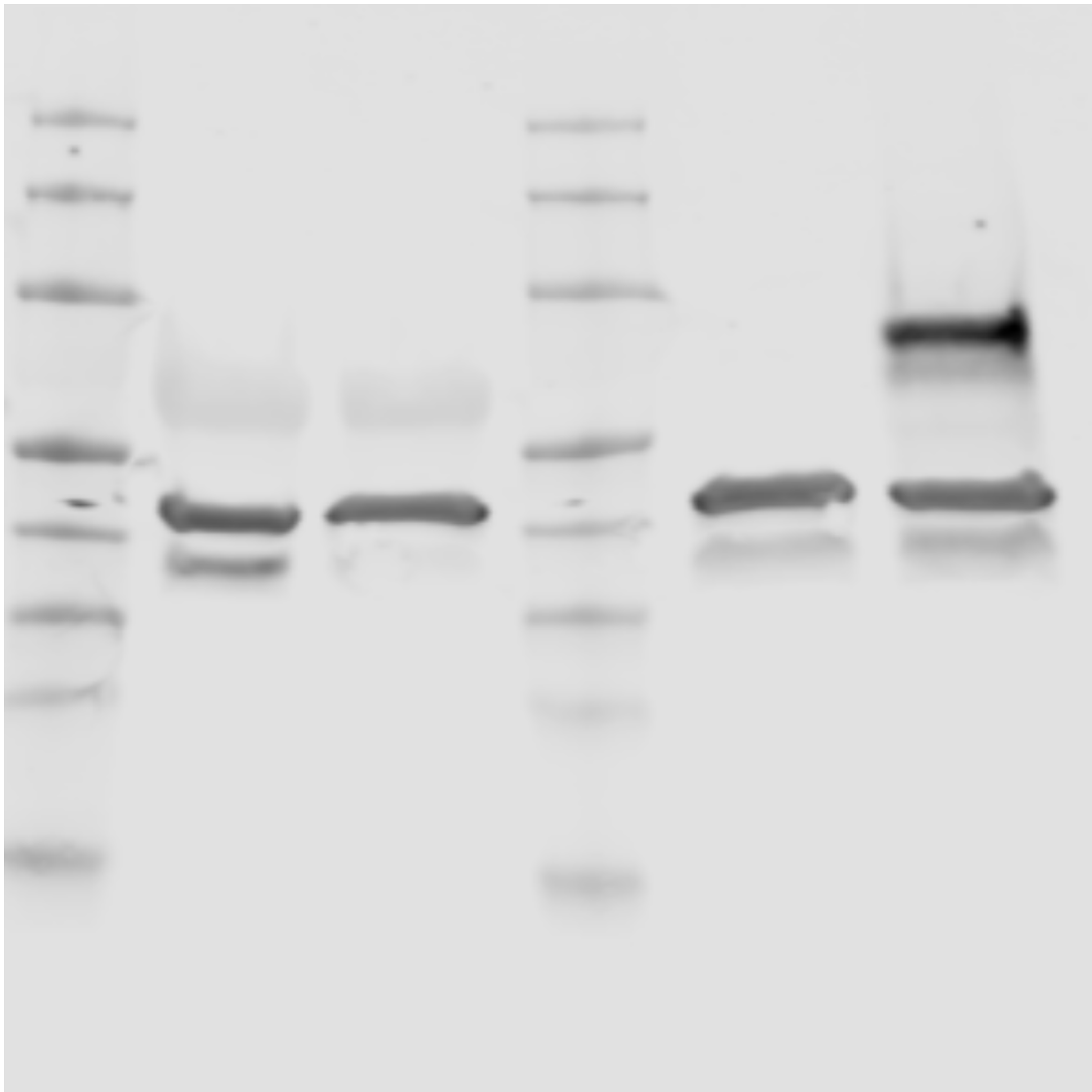

Supplementary Figure S1: Full-length blot of Figure 3A. From left to right, lanes 1-3 are from a separate experiment not included in Figure 3A. Lane 4 contains ladder, lane 5 contains non-target control transfected BEAS-2B:TET-MR1GFPs, and lane 6 contains Stx16-RFP transfected BEAS-2B:TET-MR1GFPs.

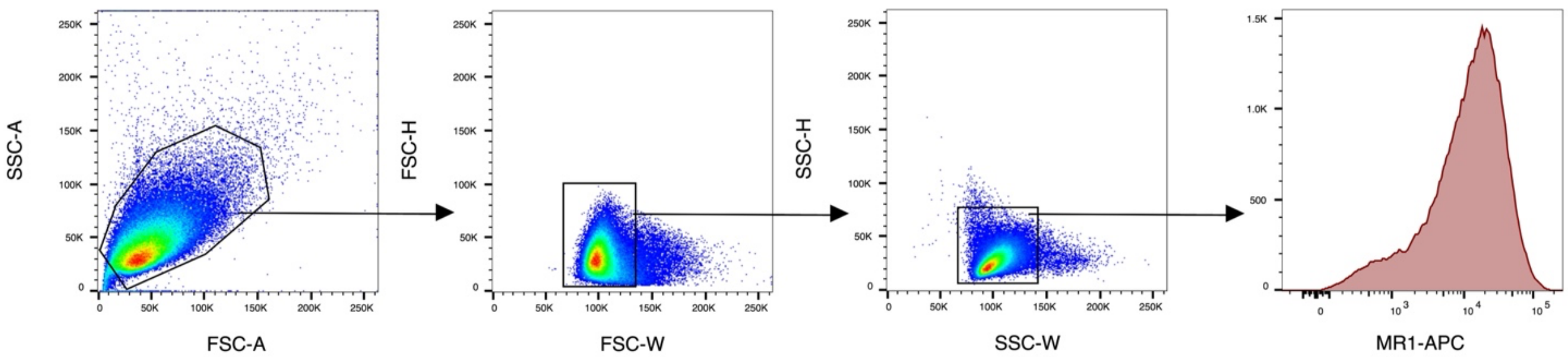

Supplementary Figure S2: Gating strategy for MR1 surface staining.
